# Kinase inhibitors in thyroid cancers

**DOI:** 10.1530/EO-22-0062

**Published:** 2023-01-16

**Authors:** Vineeth Sukrithan, Prachi Jain, Manisha H Shah, Bhavana Konda

**Affiliations:** 1 Division of Medical Oncology, Department of Internal Medicine, The Ohio State University and Arthur G James Cancer Center, Columbus, Ohio, USA

**Keywords:** sorafenib, lenvatinib, selpercatinib, dabrafenib, cabozantinib, thyroid, medullary thyroid cancer, anaplastic thyroid cancer, differentiated thyroid cancer

## Abstract

**Objective:**

The treatment landscape for thyroid cancers has changed rapidly with the availability of kinase inhibitors against VEGFR, BRAF, MEK, NTRK, and RET. We provide an up-to-date review of the role of kinase inhibitors in thyroid cancer and discuss upcoming trials.

**Design & Methods:**

A comprehensive review of the available literature describing kinase inhibitors in thyroid cancer was performed.

**Results and Conclusions:**

Kinase inhibitors have become the standard of care for patients with metastatic radioactive iodine-refractory thyroid cancer. Short-term treatment can re-sensitize differentiated thyroid cancer to radioactive iodine, thereby potentially improving outcomes and sparing toxicities associated with the long-term use of kinase inhibitors. The approval of cabozantinib as salvage therapy for progressive radioactive iodine-refractory differentiated thyroid cancer following failure with sorafenib or lenvatinib adds to the available armamentarium of active agents. Vandetanib and cabozantinib have become mainstay treatments for metastatic medullary thyroid cancer regardless of *RET* mutation status. Selpercatinib and pralsetinib, potent and selective receptor kinase inhibitors with activity against RET, have revolutionized the treatment paradigm for medullary thyroid cancers and other cancers with driver mutations in *RET*. Dabrafenib plus trametinib for* BRAF* mutated anaplastic thyroid cancer provides an effective treatment option for this aggressive cancer with a dismal prognosis. In order to design the next generation of agents for thyroid cancer, future efforts will need to focus on developing a better understanding of the mechanisms of resistance to kinase inhibition including bypass signaling and escape mutations.

## Introduction

Differentiated thyroid cancers (DTCs) represent the most common type of thyroid cancers (85%), followed by medullary thyroid cancers (MTCs) and anaplastic thyroid cancers (ATCs), representing 2 to 8% and 1%, respectively (Porter & Wong 2020). Localized DTCs, comprising papillary thyroid cancer (PTC), follicular thyroid cancer (FTC), and Hurthle cell thyroid cancer, have an excellent 10-year prognosis of about 95% (Ganly *et al.* 2015). Patients with unresectable, advanced, or refractory DTC and MTC have median 10-year survival rates of around 40–42% (Durante *et al.* 2006, Tuttle *et al.* 2010, Giunti *et al.* 2013). Poorly DTCs (PDTCs) represent a subset with features that are in between DTC and ATC. The incidence is around 6% of all cases; in patients with distant metastases, 10-year survival is around 31% (Ibrahimpasic *et al.* 2014, Fagin & Wells 2016). ATCs are the most aggressive type of thyroid cancers and are historically associated with a median survival of only 3–6 months (Lim *et al.* 2012). DTCs are mostly diagnosed in the isolated or loco-regionally advanced stage and rarely with metastasis (10%). The standard of care for either localized or metastatic DTC consists of thyroidectomy with or without lymph node dissection, long-term thyroid-stimulating hormone-suppressive levothyroxine, and adjuvant radioactive iodine (RAI) for high-risk or residual disease as well for distant metastases. Between 25 and 50% of patients with advanced or metastatic DTC develop resistance to RAI (RAI-R). These cases are associated with a median 10-year survival of 10–20% due to their aggressiveness and limited therapeutic options (Durante *et al.* 2006, Anderson *et al.* 2013). Kinase inhibitors (KIs) have become the standard of care for progressive, symptomatic metastatic DTC or RAI-R thyroid cancers and have been shown to improve progression-free survival (PFS), but at the cost of some treatment-related toxicity.

While localized MTCs are surgically curable, with a 10-year survival rate of 96%, a considerable number of patients (13%) have distant metastasis at the time of diagnosis (Roman *et al.* 2006, Wells *et al.* 2015). This is associated with a poor median survival of around 3 years, for which multitargeted receptor KIs are the mainstay of therapy. However, a statistically significant overall survival (OS) advantage has yet to be demonstrated with these therapies (Boostrom *et al.* 2009, Wells *et al.* 2012, Elisei *et al.* 2013).

ATC is an extremely aggressive cancer, associated with a median OS of 4 months, a 6-month OS of 35%, and disease-specific mortality of 98–99% (Maniakas *et al.* 2020). ATCs account for up to 50% of thyroid cancer-related deaths (Are & Shaha 2006, Smallridge *et al.* 2009, Bible *et al.* 2021). ATCs are often diagnosed at locoregionally advanced stages, invading adjacent structures or with distant metastasis, making them surgically inoperable. However, the advent of novel targeted therapies that act on mitogen-activated protein kinase (MAPK) signaling has led to improved survival in patients with ATC (Maniakas *et al.* 2020). The resources available for the treatment of advanced or metastatic thyroid cancers continue to expand with the availability of novel therapeutics (Porter & Wong 2020). The role of immune checkpoint blockade is emerging with robust evidence of efficacy in ATC. Here, we review the role of KIs as therapeutic options in advanced or metastatic thyroid cancers, either as single agents or in combination with other therapies.

## Molecular targets in thyroid cancer

### Differentiated thyroid cancer

Alterations in *BRAF, RET, RAS, PIK3CA,* and *PTEN* activate the MAPK and phosphoinositide 3-kinase (PI3K) pathways leading to the pathogenesis of many DTCs. PTC is the most common DTC (80%), wherein the common driver mutations are the *BRAF V600E* mutation (50%), followed by *RAS* mutation (15%) and* RET* fusions. FTC is characterized by the presence of either* RAS* (40–50%)* or PAX8/PPAR* fusion oncogenes (Nikiforova *et al.* 2003, Elisei *et al.* 2008, Howell *et al.* 2013).

### Poorly differentiated thyroid cancer

Compared to DTC, there is an increased prevalence of *TERT* promotor mutations (33–40%), *EIF1AX* mutations (10%), and *SWI/SNF* (6%) mutations in cases of PDTC, representing pathways of clonal evolution from slow-growing DTCs to a more aggressive phenotype closer in behavior to ATCs.

### Anaplastic thyroid cancer

The most common mutations found in ATC are *TP53* (up to 70%), *BRAF* (20–45%), and *RAS* (20–40%) (Bonhomme *et al.* 2017, Bowles *et al.* 2018). Mutations in the *SWI/SNF* components are seen in 18–36% of cases. *TERT* promoter mutations are seen in 43–73% of cases (Kunstman *et al.* 2015, Landa *et al.* 2016, Pozdeyev *et al.* 2018).

### Medullary thyroid cancer

MTC can be either familial (25%) or sporadic (75%). Virtually, all familial cases (>98%) have germline *RET* mutations (Elisei *et al.* 2019). A large study of the mutational landscape of 208 cases of sporadic MTCs showed a predilection for *RET* mutations in 56%, followed by *RAS* mutations in 24.3% (Ciampi *et al.* 2019).

## Kinase inhibitors for re-differentiation of radioactive iodine refractory differentiated thyroid cancer

### Re-sensitization to RAI therapy through BRAF and/or MEK inhibition

RAI is the cornerstone of treatment for metastatic DTCs. RAI refractory tumors are defined as tumors that show no uptake of RAI in the post-RAI-therapy scan. There are other ways to define RAI-R tumors, including tumors that show RAI uptake on initial scans, but progress within a relatively short time interval (12–14 months). Finally, it remains controversial whether patients subjected to more than 600 mCi of RAI should be considered as having RAI-R (Haugen *et al.* 2016). RAI-R tumors, when treated with conventional methods, have a poor outcome with 10-year survival as low as 10% (Gild *et al.* 2018).

KIs have been approved for progressive or advanced thyroid cancers, but the eventual emergence of resistance and treatment-related toxicities are some important limiting factors. Hence, re-sensitizing RAI-R thyroid tumors to RAI is an area of active clinical and research interest (Fullmer *et al.* 2021).

Activating mutations in driver pathways, such as *BRAF* and *NRAS* mutations in the MAPK pathway, lead to hyperactive signaling, which decreases the expression of sodium/iodide symporter and reduces iodine uptake (Liu *et al.* 2007). Pre-clinical evidence of restored RAI uptake with BRAF inhibition *in vivo* led to a clinical study of MEK inhibition with selumetinib for the purpose of re-sensitization. Four weeks of treatment resulted in a clinically meaningful increase in RAI uptake in 40% patients (8 of 20) with metastatic RAI-R DTC including 100% of *NRAS*-mutated subjects (5 of 5) and 44% of *BRAF*-mutated patients (44%, 4 of 9). Of the eight patients treated with RAI, 63% (5 of 8) had confirmed partial responses (PRs) (Chakravarty *et al.* 2011, Ho *et al.* 2013). A report by Rothenberg *et al.* demonstrated the clinical efficacy of the selective BRAF inhibitor, dabrafenib, in inducing RAI uptake in *BRAF V600E*-mutated unresectable or metastatic RAI-R PTC. In this cohort, 60% (6 of 10) had increased RAI uptake post-treatment with dabrafenib. Two of these six patients (33%) had a PR (Rothenberg *et al.* 2015). A pilot trial of vemurafenib, a BRAF inhibitor, was conducted in 12 patients with *BRAF-*mutated RAI-R DTC. Four of the 10 evaluable patients had uptake meeting thresholds for treatment; PRs were seen in 50% (2 of 4 patients), with the other 2 patients having the stable disease (SD) at the 6-month mark (Dunn *et al.* 2019).

A retrospective review at MD Anderson of 13 patients on long-term BRAF or a MEK inhibitor with RAI uptake reported results from 9 patients (3 *RAS*, 5 *BRAF*,1 wild type) who subsequently underwent RAI treatment (Jaber *et al.* 2018). Responses were seen in 40% (2 of 5) *BRAF*-positive patients and 33% (1/3) *RAS*-mutant patients with the rest having stable disease. Another retrospective study reported six patients (three with *NRAS* mutation and three with *BRAF V600E* mutation) who were treated with either a MEK inhibitor or the combination of a BRAF and MEK inhibitor for *RAS-* and *BRAF-*mutated patients, respectively (Iravani *et al.* 2019). One *NRAS-* and all *BRAF V600E*-mutated cases demonstrated restoration of RAI uptake and proceeded to RAI therapy. The *NRAS*-mutated case (1 of 1) and 66% (2 of 3) of patients with a *BRAF V600E* mutation demonstrated PR beyond a 3-month follow-up.

These studies have led to the use of KIs to re-sensitize metastatic unresectable RAI-R thyroid tumors to further doses of RAI, thereby salvaging this valuable treatment option.

MERAIODE is a prospective multicentric phase 2 trial of the combination of dabrafenib and trametinib for re-differentiation in patients with *BRAF V600E* mutations and trametinib alone in *RAS*-mutated RAI-R metastatic DTC with RECIST progression within 18 months (Dunn *et al.* 2019). Among 21 evaluable patients with a *BRAF* mutation, at 6 months, the rate of PR was 38%, 52% had SD, and 10% had progressive disease (PD). In a separate cohort of 10 evaluable *RAS*-mutated patients, the rate of PR was 20% with SD in 70% and PD in 10% (Leboulleux *et al.* 2021).

ASTRA was a phase 3 trial of adjuvant RAI vs RAI with a MEK inhibitor (selumetinib) in patients with high-risk DTC after resection (Ho *et al.* 2022). Patients with high-risk disease were randomized in a 2:1 ratio to either selumetinib or placebo for 5 weeks. A single dose of adjuvant RAI therapy was administered on treatment days 29 to 31. The trial failed to show a statistically significant difference in the complete response rate at 18 months (OR = 1.07; *P* = 0.82). Treatment-related grade ≥3 adverse events (AEs) were reported in 25 of 154 patients (16%) with selumetinib while there were none with placebo. An acneiform rash was seen in 7% of patients.

Unlike agents that target the MAPK pathway, various other therapies including retinoids and multitargeted KIs such as sorafenib and lenvatinib have failed to show clinically meaningful response in re-sensitizing RAI-R thyroid cancers to iodine (Hoftijzer *et al.* 2009).

### Re-sensitization to RAI therapy through RET and NTRK inhibition

There is emerging evidence that DTCs with fusions in *RET* and *NTRK* are amenable to re-differentiation through therapies that target the driver alterations. A case series reported re-differentiation in two out of three consecutive patients with *NTRK*-rearranged RAI-resistant thyroid cancers treated with the NTRK inhibitor larotrectinib (Groussin *et al.* 2020, 2022). A pediatric case series of two patients with *NTRK* and *RET* fusion treated with larotrectinib and selpercatinib reported decreased tumor sizes and restored RAI uptake (Lee *et al.* 2021). Another case series reported two pediatric cases and documented a case of re-differentiation with selpercatinib targeting a *RET* fusion gene in metastatic RAI-resistant PTC (Groussin *et al.* 2021). Interestingly, in one of the pediatric case reports of metastatic papillary thyroid carcinoma, larotrectinib was shown to induce redifferentiation when given in the neoadjuvant setting prior to RAI, thereby achieving an improved outcome (Waguespack *et al.* 2022*b*). Prospective studies using such targeted agents are needed to confirm these findings and further expand the options to restore radioiodine uptake in RAI-resistant thyroid cancers.

## Kinase inhibitors for advanced radioactive iodine refractory differentiated thyroid cancer

### VEGFR-targeted kinase inhibitors

While sorafenib is approved as a frontline treatment, lenvatinib has efficacy in the first- or second-line setting. Cabozantinib (brand name: Cabometyx) has been approved in the second-line setting for patients with RAI-R DTC who have progressed following up to two prior vascular endothelial growth factor receptor (VEGFR)-targeted therapies.

Initial phase 2 trials of sorafenib in DTC showed median PFS ranging from 58 weeks to 84 weeks with a manageable toxicity profile, paving the way for larger phase 3 trials (Gupta-Abramson *et al.* 2008, Ahmed *et al.* 2008, Brose *et al.* 2009, Hoftijzer *et al.* 2009, Kloos *et al.* 2009). In 2013, the US Food and Drug Administration (FDA) approved sorafenib in the first line for RAI-R advanced or metastatic DTC based on data from the phase 3 decision trial, which showed significantly improved median PFS of 10.8 months vs 5.8 months in the placebo group (HR, 0·59; *P* < 0.0001) (Brose *et al.* 2014). This benefit was observed regardless of *BRAF* or *RAS* mutation. While improved PFS was supportive of the use of sorafenib as a treatment option in RAI-R DTC, responses occurred in only 12% of subjects. The most common AEs in the sorafenib arm were hand–foot skin reaction, diarrhea, alopecia, rash/desquamation, fatigue, weight loss, and hypertension.

Lenvatinib is a multitargeted KI, which inhibits VEGFR 1–3 (VEGFR1–3), fibroblast growth factor receptors 1–4 (FGFR1–4), RET, c-KIT, and platelet-derived growth factor receptor α (PDGFRα). In 2015, the FDA approved lenvatinib for RAI-refractory DTC with disease progression, including those with prior exposure to one tyrosine kinase inhibitor (TKI), based on the phase 3 trial (SELECT). This study showed a significantly improved median PFS of 18.3 vs 3.6 months (HR: 0.21; *P* < 0.001) in addition to an increased response rate of 64.8% vs 1.5% (OR 28.87; *P* < 0.001) (Schlumberger *et al.* 2015). After adjusting for crossover effects, there was a trend toward improved OS (HR, 0.62; 95% CI, 0.40–1.00; *P* = 0.05). The response was maintained regardless of the patient’s *BRAF* or *RAS* mutation status. Commonly reported AEs were hypertension, diarrhea, fatigue/asthenia, and decreased appetite.

Cabozantinib, a multikinase inhibitor targeting VEGFR2, MET, AXL, and RET, was approved by the FDA in 2021 for RAI-R DTC patients who progressed after up to two prior VEGFR-targeted therapies, based on data from the phase 3 COSMIC-311 study (Brose *et al.* 2021*b*). The trial demonstrated significantly improved median PFS for the cabozantinib group over the placebo group of 11 vs 1.9 months (HR, 0.22; 95% CI, 0.15–0.31) (Brose *et al.* 2021*a*,*b*, Capdevila *et al.* 2021). Although it did not meet the co-primary endpoint of ORR – 15% for cabozantinib vs 0% for placebo (*P* = 0.0281) – a favorable OS trend was observed (HR 0.54, 95% CI 0.27–1.11). The benefit in PFS was seen across all subgroups, either with prior sorafenib or lenvatinib treatment alone or after progression on both. Cabozantinib is therefore a promising agent for salvage in patients who have progressed on the prior-approved VEGFR-targeted therapies. Common AEs were diarrhea, palmar-plantar erythrodysesthesia, fatigue, hypertension, and stomatitis.

Sunitinib, a multi-targeted inhibitor of VEGFR, RET, and PDGFR, was studied in a single-center phase 2 study among 23 patients who had received at least one dose of RAI. Six (26%) patients achieved a PR, and 13 (57%) had SD with a median PFS of 8 months (Bikas *et al.* 2016). Another phase 2 study that enrolled 71 patients showed sunitinib to be active in metastatic DTC and MTC, with ORR of 22 and 38.5%, respectively, and OS of 26.4 and 29.4 months, respectively (Ravaud *et al.* 2017). The study did not support the proposed dose of 50 mg/day due to the occurrence of pronounced side effects. Overall, there is limited evidence of the clinical activity of sunitinib in advanced thyroid cancer (Cohen *et al.* 2008, Carr *et al.* 2009, Gómez-Sáez 2016).

Pazopanib is another multikinase inhibitor targeting VEGFR, PDGFR, and KIT, which showed promising activity and manageable toxicity in a phase 2 trial of a pre-treated group of 60 patients with progressive RAI-R DTC (Bible *et al.* 2010). The trial demonstrated an ORR of 37%, a median PFS of 11.4 months, and a median OS of 2.6 years. Hypertension, fatigue, and neutropenia were commonly observed grade 3–5 toxicities.

Axitinib, a potent selective inhibitor of VEGFR*,* was shown to have activity in advanced thyroid cancer, across all histologic subtypes including RAI-R DTC in two phase 2 trials. The two trials reported an ORR of 38 and 35%, PFS of 15 and 16.1 months, and duration of response of 21 and 17.2 months, respectively. The median OS was 35 and 27.2 months, respectively (Cohen *et al.* 2014, Locati *et al.* 2014). Commonly reported AEs were fatigue, diarrhea, nausea, anorexia, hypertension, and weight loss.

### mTOR-targeted kinase inhibitors

Everolimus, an mTOR inhibitor, was shown to have limited efficacy in the second-line setting post-progression on a prior TKI in a trial of 50 subjects. ORRs of 3, 10, and 14% were reported for DTC, MTC, and ATC, respectively (Hanna *et al.* 2018). Another multi-institutional phase 2 study showed improved PFS benefit with the addition of everolimus to sorafenib in a cohort with RAI-R Hurthle cell cancer (24.7 vs 10.9 months), but similar OS (Sherman *et al.* 2021).

### ALK-targeted kinase inhibitors

Anaplastic lymphoma kinase (*ALK*)-targeted therapies, such as crizotinib, ceritinib, brigatinib, loratinib, and alectinib, are well-established FDA-approved treatment options in *ALK*-translocated non-small cell lung cancer (NSCLC). Genomic profiling of thyroid cancers has led to the discovery of *ALK* rearrangements in around 2% of PTCs, including a novel *ALK* fusion product,STRN-ALK, that is sensitive to available ALK inhibitors (Kelly *et al.* 2014). A case study showed the clinical efficacy of lorlatinib in a refractory DTC harboring *EML4-ALK* fusion. The patient had a PR that was ongoing at 7 months, with no treatment-related AEs (Aydemirli *et al.* 2021).

### BRAF/MEK-targeted kinase inhibitors

The role of BRAF inhibition with driver mutations at the *BRAF V600* locus is a topic of active study. A clinically meaningful response to vemurafenib in *BRAF V600E-*mutated PTC was shown in a nonrandomized phase 2 trial, with PR rates of 38.5 vs 27.3% in VEGFR-TKI naïve and VEGFR TKI experienced cohorts, respectively. The median PFS was 18.2 and 8.9 months, respectively (Brose *et al.* 2016).

A randomized phase 2 trial of dabrafenib alone or dabrafenib and trametinib was conducted in 53 cases of *BRAF*-mutated papillary thyroid carcinoma (Busaidy *et al.* 2022). Patients with refractory DTC with PD within 13 months were eligible. The primary outcome of the response rate by per-protocol modified RECIST was not statistically significantly different (42 vs 48%, *P* = 0.67) in the single and combination arms, respectively. The objective response rate by RECIST 1.1 was 35% (9 of 26) with dabrafenib and 30% (8 of 27) with dabrafenib and trametinib. The median PFS was numerically prolonged in the combination arm but did not reach statistical significance (10.7 vs 15.1 months, *P* = 0.65). In a small subset of 11 patients who were pre-treated with multikinase inhibitors, the median PFS with dabrafenib was 3.7 and 18.8 months with combination therapy (*P* = 0.01)

### RET-targeted kinase inhibitors

Large-scale genomics studies indicate that *RET* rearrangements occur in around 7% of PTCs (Cancer Genome Atlas Research Network 2014). In a trial of the novel selective RET inhibitor selpercatinib that reported data from 19 patients with *RET* fusion-positive, previously treated thyroid cancer, objective responses were seen in 79% (Wirth *et al.* 2020). The activity was observed across the board in thyroid cancers including anaplastic carcinomas, and across different fusion partners. Pralsetinib, another potent, selective RET inhibitor, was also shown to have clinically meaningful activity in *RET* fusion-positive thyroid cancer with an ORR of 89% (8 of 9 patients) (Subbiah *et al.* 2021*a*, Mansfield *et al.* 2022).

### NTRK-targeted kinase inhibitors

Chromosomal rearrangements of *NTRK* genes are oncogenic driver mutations seen in around 3% of patients with thyroid cancer (Solomon *et al.* 2020*b*). *NTRK* fusions occur across a range of pediatric cancers including infantile fibrosarcoma and secretory breast cancer in which such fusions are nearly pathognomonic (Blauel & Laetsch 2022). Pooled data of thyroid cancers treated with the NTRK inhibitor larotrectinib from three trials (NCT02576431, NCT02122913 and NCT02637687) showed that the objective response rates were 86% for DTC and 29% among those with ATC (Waguespack *et al.* 2022*a*). In an integrated analysis of three phase 1/2 trials of patients with driver *NTRK* fusions treated with entrectinib, among 13 patients with thyroid cancer, responses were seen in 54% (7 of 13) and a median PFS of 19.9 months was reported (Demetri *et al.* 2022).

Select clinical trials of KIs in DTC with associated clinical results are summarized in Table 1.

**Table 1:** Select clinical trials of kinase inhibitors (KIs) in differentiated thyroid cancer.

Agent	Molecular targets	Trial phase	Subject population	Outcome		
				Median PFS (months) and/or DoR	ORR (%)	Reference
**RAI + BRAF/MEK inhibitors (use of KIs as redifferentiating agents)**						
Selumetinib + RAI	MEK	Prospective	RAIR DTC (follicular thyroid cancer)	NA	62% (5/8)	Ho *et al.* 2013
Dabrafenib + RAI	BRAF	Prospective	BRAF V600E RAIR DTC	NA	33% (2/6)	Rothenberg *et al.* 2015
Selumetinib + RAI vs RAI	MEK	Phase 3	Adjuvant treatment in high-risk DTC	NA	40% (62/155)	Ho *et al.* 2022
**VEGFR KIs in DTC**						
Sorafenib	VEGFR 1–3, PDGFR, RET, cKIT, BRAF	Phase 3	Advanced RAIR DTC	10.8 vs 5.8 Placebo	12.2% (24/196)	Brose *et al.* 2014
Lenvatinib	VEGFR 1–3, FGFR 1–4, PDGFR, RET, c-KIT	Phase 3	Advanced RAIR DTC	18.3 vs 3.6 Placebo	64.8% (169/261)	Schlumberger *et al.* 2015
Cabozantinib	VEGFR, MET, RET, AX	Phase 3	Advanced RAIR DTC	11 vs 1.9 Placebo	15% (10/67)	Brose *et al.* 2021*a*; Brose *et al.* 2021*b*
**BRAF/MAPK inhibitors in DTC**						
Dabrafenib + trametinib vs dabrafenib	BRAF/MEK	Phase 2	RAIR PTC	D + T: 15.1 (11.7–NR) D: 11.4 (3.8–NR)	D + T: 35% (9/26) D: 45% (10/22)	Busaidy *et al.* 2022
Vemurafenib	BRAF V600E	Phase 2	Advanced (metastatic/unresectable) RAIR PTC (KI Naïve)	18·2	38.5% (10/26)	Brose *et al.* 2016
			Advanced (metastatic/unresectable) RAIR PTC (prior exposure to KI)	8.9	27.3% (6/22)	
**NTRK inhibitors in NTRK fusion positive DTC/ATC**						
Larotrectinib	NTRK1, NTRK2, NTRK3 fusion inhibitor	Phase 1/2	Advanced DTC	DoR of 24 months = 84% PFS at 24 months = 84%	86% (18/21)	Waguespack *et al.* 2022*a*
			Anaplastic thyroid cancer	DoR of 12 months = 50% Median PFS = 2.2 months	29% (2/7)	
Entrectenib	NTRK1,NTRK2 ,NTRK3 fusion inhibitor	Phase 1/2	Unspecified thyroid cancer	mPFS = 19.9 months mDoR = 13.2 months	54% (7/13)	Demetri *et al.* 2022
**RET inhibitors in RET-fusion positive DTC/ATC**						
Selpercatinib	RET inhibitor	Phase 1/2	Multiple histologic types (papillary thyroid cancer, Hurthle cell, poorly differentiated, ATC)	PFS at 12 months = 64%	79% (15/19)	Wirth *et al.* 2020
Pralsetinib	RET inhibitor	Phase 1/2	RET fusion positive thyroid cancer previously treated	mDoR = 17.5 months mPFS = 19.4 months	86% (18/21)	Mansfield *et al.* 2022

ATC, anaplastic thyroid carcinoma; DoR, duration of response; DTC, differentiated thyroid carcinoma; MTC, medullary thyroid carcinoma; NR, not reached; ORR, overall response rate; PFS, progression-free survival; RAI-R, radioactive iodine-refractory.

## Kinase inhibitors in anaplastic thyroid cancer

ATCs are one of the most aggressive malignancies with a historical median OS of around 4 months (Lin *et al.* 2019). Recent advances in understanding the molecular biology of these cancers have begun to translate to better survival. Unique characteristics such as a prevalence of *BRAF* mutations, high PD-L1 staining, and increased neo-angiogenesis make ATC susceptible to novel molecular targeted therapies such as BRAF inhibition and VEGFR inhibitors in combination with immunotherapy (Bastman *et al.* 2016, Chintakuntlawar *et al.* 2017, Zwaenepoel *et al.* 2017, Bowles *et al.* 2018, Rosenbaum *et al.* 2018, Cantara *et al.* 2019, Zhang *et al.* 2019). A single-center retrospective study of OS by treatment era in stage 4-C ATC patients (*n* = 253) from 2000 to 2019 showed an improvement in the rate of survival to 1 year: 35% in the pre-2013 group, 47% in the 2014–2016 cohort, and 59% in the 2017–2019 cohort (hazard ratio: 0.5) (Maniakas *et al.* 2020). The importance of rapid molecular genotyping for the immediate initiation of targeted therapies against *BRAF, NTRK,* and other KIs cannot be overstated.

Dabrafenib plus trametinib was approved by the FDA in 2018 for patients with locally advanced or metastatic *BRAF V600E*-mutated ATC with no satisfactory locoregional treatment options, based on a clinical trial that showed a high overall response rate (69%), prolonged duration of response, and prolonged survival with manageable toxicity (Subbiah *et al.* 2018). Further updated analysis showed continued clinically meaningful benefits with a median PFS and OS of 6.7 and 14.5 months, respectively (Subbiah *et al.* 2022). Neoadjuvant treatment of unresectable *BRAF*-mutated ATCs with BRAF/MEK inhibitors is associated with significant, rapid therapeutic responses, rendering them surgically operable in many cases (Cabanillas *et al.* 2018, Wang *et al.* 2019, Maniakas *et al.* 2020).

Multi-receptor KIs do not have a role in ATC as single agents. Sorafenib and pazopanib failed to show any treatment efficacy in ATC (Garon *et al.* 2015, Villaruz & Socinski 2016, Villaruz *et al.* 2019). Lenvatinib as a single agent was studied in a multicenter single-arm phase 2 trial. Three percent (1 of 34) had a PR and 50% (17 of 34) of patients achieved SD (≥5 weeks). Even though more than half of the evaluable patients experienced some degree of tumor shrinkage and three patients experienced a >30% tumor reduction, the response duration was short-lived: the median PFS and OS were 2.6 and 3.2 months, respectively (Wirth *et al.* 2021). There is promising evidence of added efficacy with the combination of lenvatinib with immune-checkpoint inhibitors in ATC, which is summarized in the section ‘combination of kinase inhibitors with immune checkpoint inhibitors’.

In a phase 1/2 study, a deep and durable PR was reported in one patient with widely metastatic ATC, harboring a *CCDC6-RET* gene fusion, treated with the RET inhibitor LOXO-292 following progression on conventional treatment (Dias-Santagata *et al et al.* 2020). Further studies are needed to establish the benefit of RET inhibitors in this cohort of *RET-*harboring, progressive ATC.

## Kinase inhibitors in medullary thyroid cancer

KIs have become the mainstay treatment for advanced, progressive, symptomatic, and metastatic MTCs. Vandetanib (RET, VEGFR2/3, EGFR) and cabozantinib (brand name: Cometriq) are multitargeted KIs, approved in 2011 and 2012, respectively, based on data from randomized, placebo-controlled phase 3 trials for unresectable or metastatic MTC. In the ZETA trial, an ORR of 44% and PFS of 30.5 months were reported with vandetanib when compared to 19.3 months for placebo. The EXAM trial was another phase 3 trial that showed an ORR of 28% and PFS of 11.2 months with cabozantinib when compared to 4 months with placebo (Wells *et al.* 2012, Elisei *et al.* 2013). These trials did not pre-specify analysis of clinical outcomes by *RET* mutation status, and it is not currently known if clinical efficacy depends on the presence of *RET* mutations. There are numerous differences in the patient demographics of these studies that preclude inter-trial comparisons. There are no trials of direct head-to-head comparisons of vandetanib with cabozantinib and KI selection depends on individual patient characteristics. Common side effects with vandetanib include diarrhea, rash, acne, fatigue, nausea, hypertension, headache, vomiting, and photosensitivity. Vandetanib carries a black box warning of QT interval prolongation, torsade de pointes, and sudden death. Rare but serious AEs were reported, including reversible posterior leukoencephalopathy and Stevens–Johnson syndrome. The most commonly reported adverse drug reactions with cabozanitinib are diarrhea, stomatitis, palmar-plantar erythrodysesthesia syndrome, decreased weight, decreased appetite, nausea, fatigue, oral pain, hair color changes, dysgeusia, hypertension, abdominal pain, and constipation. It comes with a black box warning of hemorrhage, perforations, and fistulas. Other serious AEs include thromboembolic events, poor wound healing, osteonecrosis of the jaw, and reversible posterior leukoencephalopathy syndrome.

A meta-analysis studying KIs in MTC that included 1 phase 4 study, 2 phase 3 trials, 20 phase 1 or 2 studies, and 10 retrospective-observational studies showed that grade 3 AEs led to treatment discontinuation in 39.6 and 66.7% of patients treated with vandetanib and cabozantinib, respectively. A risk–benefit discussion with patients is important for decision-making to ascertain the best timing to start KIs (Efstathiadou *et al.* 2021). Case reports have shown a possibility of a cessation trial in patients with documented SD, but further prospective clinical trials would be needed before a discussion on the cessation of KIs (Brandenburg *et al.* 2021).

Selpercatinib is a selective RET KI with activity against diverse* RET* alterations, including the acquired gatekeeper resistance mutation at residue V804. Similar high response rates were seen in TKI-naive (73%) and pre-treated patients with *RET*-mutated MTC (69%) (Wirth *et al.* 2020). Pralsetinib is another RET-specific TKI with a treatment efficacy (ORR of 74% in TKI-naïve patients and 60% in those with prior TKI exposure) and side-effect profile similar to selpercatinib (Subbiah *et al.* 2021*a*). These agents are well tolerated with only 2% of patients on the trial of selpercatinib discontinuing medication due to toxicity. The most common grade 3/4 AEs were hypertension (21%), increased alanine aminotransferase level (11%), increased aspartate aminotransferase level (9%), hyponatremia (8%), and diarrhea (6%). There were no treatment-related deaths. The emergence of solvent-front mutations in *RET* at the G810 locus is associated with resistance to selective *RET* inhibitors; next-generation agents with putative activity against escape mutations are under active investigation (NCT05241834). Select clinical trials of KIs with associated clinical results in MTC are summarized in Table 2.

**Table 2:** Select trials of kinase inhibitors in medullary thyroid cancer.

Agent	Molecular targets	Trial phase	Subject population (# of prior lines)	Outcome		
				Median PFS (months)	ORR (%)	Reference
Cabozantinib	VEGFR, MET, RET, AX	Phase 3	MTC	11.2 vs 4	28% (58/208)	Elisei *et al.* 2013
Vandetanib	VEGFR, EGFR, c-KIT, RET	Phase 3	MTC	30.5 vs 19.5	44% (101/231)	Wells *et al.* 2012
Selpercatinib	RET	Phase 1/2	MTC: treatment naive	NR	73% (64/88) 69% (38/55)	Wirth *et al.* 2020
			MTC: previously treated			
Pralsetinib	RET	Phase 1/2	MTC: treatment naive	NR	71% (15/21) 60% (33/55)	Subbiah *et al.* 2021*a*
			MTC: previously treated			

MTC, medullary thyroid carcinoma; NR, not reached; ORR, overall response rate; PFS, progression-free survival.

For patients with *RET*-mutant MTC, the advent of potent and selective RET inhibitors has led to a paradigm shift in treatment due to the highly improved risk–benefit profile with these novel agents when compared to older non-selective KIs, such as vandetanib and cabozantinib. For patients with vascular or bowel tumor involvement or elevated risk of thrombosis or hemorrhage, selective RET inhibitors open up treatment avenues that were otherwise closed due to unacceptably elevated risks with earlier agents. The efficacy of these agents in patients who have progressed on novel RET-selective agents is currently unknown and is an area of active research.

## Combination of kinase inhibitors with immune checkpoint inhibitors

There is emerging evidence that angiogenic signaling in the tumor and microenvironment contributes to immune exclusion and primary resistance to checkpoint blockade. In a thyroid cancer tissue banking study, PD-1 and PD-L1 expression were noted in 20 and 64% of DTC samples, respectively (Bastman *et al.* 2016). FoxP3, PD-1 CD8, and PD-1 CD4 T cells were all noted to be abundant in DTC. Thus, immune evasion plays a vital role in the pathogenesis of DTC; subsequently, low response rates are encountered in single-agent immune checkpoint inhibitor treatment (ORR: 9%) (Mehnert *et al.* 2019). Currently, checkpoint inhibition is not approved for thyroid cancers. Pembrolizumab may still be used in advanced thyroid cancers with a high mutational burden or with mutations in DNA mismatch repair genes due to its approval in a tumor-agnostic fashion in this subset of patients.

The synergistic effect of the combination of checkpoint blockade and KIs in metastatic refractory thyroid cancer was reported in a single-institution case series (Porter *et al.* 2020). A single-arm phase 2 trial of the combination of lenvatinib (20 mg) and pembrolizumab in patients with RAI-R DTC with PD within 14 months was reported (Haugen *et al.* 2020). Patients with prior exposure to VEGFR-targeted therapy were excluded. Of 29 evaluable patients, 18 (62%) had a PR and 10 (35%) had SD. The PFS at 12 months was 74%.

A phase 2 study exploring the combination of cabozantinib with nivolumab and ipilimumab in patients with RAI-R DTC and disease progression with up to two prior VEGFR-targeted therapies is currently underway (NCT03914300). In ATC, immunotherapy with spartalizumab, an anti-PD-1 agent, is associated with an ORR of 19% (Capdevila *et al.* 2020). Results from a phase 2 trial of lenvatinib and pembrolizumab in patients with ATC and poorly differentiated thyroid cancer (ATLEP) showed that the combination may have significant synergy when compared to each drug alone. Among the first 26 patients with ATC treated with the regimen, ORR after 3 months of treatment was 38.5% (10 of 26, all PR). Additionally, 57.6% (15 of 26) patients achieved SD. One patient had PD. At the 2-year survival landmark, among 16 evaluable patients, the best-observed response rate was 68.5% (11 of 16), indicating that responses tended to improve with time. The median PFS was 8.3 months and OS was 10 months (Dierks 2021).

## Mechanisms of resistance to kinase inhibition in thyroid cancer

### Resistance to BRAF/MEK-targeted KIs

The eventual development of resistance to KIs remains a major limitation in the treatment of advanced thyroid cancers. A better understanding of the biologic underpinnings of resistance is needed to develop the next generation of thyroid cancer treatments. There is emerging evidence that resistance to BRAF inhibition can occur by bypass activation of the MAPK pathway through *de novoRAS* mutations (Owen *et al.* 2019, Cabanillas *et al.* 2020). In *BRAF V600E*-mutated cells, epigenetic up-regulation of HER3 expression through an autocrine NRG1 loop has been described (Montero-Conde *et al.* 2013). Also, mutations altering cell cycle or DNA damage recognition pathways are alternate mechanisms of resistance (Duquette *et al.* 2015, Antonello *et al.* 2017). Morphological de-differentiation through activating *RAC1* (P34R) mutations and gene amplification has also been described. Epithelial to mesenchymal transformation through the amplification of the *TWIST1* transcription factor and *MET* gene amplification are other potential mechanisms. Excess *RAC1* activity leads to an increase in phosphorylated PAK1, which promotes the abnormal organization of mitotic spindles (Bagheri-Yarmand *et al.* 2021). Emerging evidence suggests that mutations in *SWI/SNF* chromatin remodeling complexes may predict resistance to re-differentiation with BRAF/MEK inhibitors; novel strategies that target this will need to be elucidated (Saqcena *et al.* 2021). Current ongoing clinical trials of KIs in DTC are summarized in Table 3.

**Table 3:** Select ongoing clinical trials of KIs for thyroid cancers (ClinicalTrials.gov).

Agent	Phase	Population	NCT
**BRAF/MAP kinase pathway inhibitors +/− RAI in DTC**			
BRAF: dabrafenib and trametinib RAS: trametinib + RAI	Phase 2	BRAF- or RAS-positive DTC with progression within 18 months	NCT03244956
Selumetinib plus RAI vs RAI alone	Phase 2	DTC with RAI avidity	NCT02393690
Selumetinib and RAI	Phase 2	DTC with progression within 12 months	SEL-I-METRY
Encorafenib and binimetinib with or without nivolumab	Phase 2	Metastatic RAI-R BRAF V600-mutant DTC	NCT04061980
Vemurafenib plus copanlisib	Phase 1	RAI-R DTC	NCT04462471
Spartalizumab in combination with MAPK pathway inhibitors	Phase 2	RAI-R DTC	NCT04544111
Dabrafenib and lapatinib	Phase 1	RAI-R/unresectable DTC	NCT01947023
Cabozantinib in combination with atezolizumab	Phase 1/2	Locally advanced or metastatic solid tumors (DTC)	NCT03170960
Cabozantinib, nivolumab, and ipilimumab	Phase 2	Advanced RAI-R DTC with progression on up to 2 VEGFR-targeted therapies	NCT03914300
Lenvatinib and pembrolizumab	Phase 2	DTC	NCT02973997
Lenvatinib and everolimus	Phase 2	Metastatic DTC with progression on lenvatinib alone	NCT03139747
Anlotinib	Phase 2	Locally advanced thyroid cancer	NCT04309136
Apatinib	Phase 3	RAI-R DTC	NCT03048877
**VEGFR inhibitors and immunotherapy combinations in ATC**			
Lenvatinib and pembrolizumab	Phase 2	Unresectable stage IVB and IVC anaplastic thyroid cancers	NCT04171622
Lenvatinib and pembrolizumab	Phase 2	Anaplastic thyroid cancers and poorly differentiated thyroid cancers	ATLEP
**RET inhibitors**			
TPX-0046	Phase 1/2	Advanced or metastatic solid tumors harboring RET mutations or alterations	NCT04161391
TAS0953	Phase 1/2	RET-altered solid tumors	NCT04683250
Selpercatinib	Phase 2	Neoadjuvant therapy in RET-altered thyroid cancer	NCT04759911
BOS172738	Phase 1	Advanced RET altered tumors	NCT03780517
LOXO-260	Phase 1	RET fusion-positive solid tumors, medullary thyroid cancer, and other tumors with RET activation refractory to selective RET inhibitors	NCT05241834
**NTRK inhibitors**			
Larotrectinib	Phase 2	Locally advanced thyroid cancers harboring NTRK fusions	NCT02576431
Larotrectinib	Phase 1	Solid tumors harboring NTRK fusion	NCT02122913
Entrectinib	Phase 2	Locally advanced papillary thyroid cancer with RET fusions	NCT02568267
Selitrectinib	Phase 1	Solid tumors harboring NTRK fusion	NCT03215511
Repotrectinib	Phase 1/2	Locally advanced/metastatic solid tumors	NCT03093116

DTC, differentiated thyroid carcinoma; RAI, radioactive iodine; RAI-R, RAI-refractory.

### Resistance to RET-targeted KIs

Treatment with novel RET-targeted KIs, selpercatinib and pralsetinib, can overcome resistance from gatekeeper mutations but, in turn, can lead to the emergence of resistance mediated by novel non-gatekeeper mutations.

Solvent-front mutations at the G810 site were first described after two patients with MTC and *RET* fusion-positive NSCLC developed resistance to selpercatinib (Solomon *et al.* 2020*a*). In-depth studies using X-ray crystallography confirmed that mutations at the C-lobe solvent-front (G810C/S/R), hinge site (Y806C/N), and the β2 strand (V738A) all led to a steric clash at the drug-binding site (Subbiah *et al.* 2021*b*).

A study analyzing post-treatment tissue and/or liquid biopsies obtained from 18 patients with resistance to a RET-selective inhibitor showed acquired *RET* mutations at the *RET G810* residue (11%), *MET* amplification (17%), or *KRAS* amplification (6%) as the most frequently occurring molecular mechanisms of resistance (Lin *et al.* 2020). Novel agents that can target solvent-front mutations at the G810 site (LOXO-260, TPX-0046, BOS172738, TAS0953) are currently being investigated (Table 3).

### Resistance to NTRK-targeted KIs

A study of larotrectinib in *TRK* fusion-positive cancers in adults and children reported patterns of resistance in a subset of patients who experienced disease progression during treatment after a documented objective response or SD for at least 6 months (*n* = 10 patients) (Drilon *et al.* 2018). Multiple plasma samples obtained upon progression in nine patients showed kinase domain mutations in the *NTRK* gene, including substitutions in the solvent-front position (*NTRK1 G595R* or *NTRK3 G623R*, 78%), gatekeeper mutations (*NTRK1 F589L*, 22%), or xDFG activation-loop mutations (*NTRK1 G667S* or *NTRK3 G696A*, 22%).

A retrospective study of 18 patients with progression on first-generation *TRK* inhibitors (larotrectinib = 13 and entrectinib = 5) showed that the majority had on-target resistance (83%, *n* = 15 of 18) compared to off-target resistance (11%, *n* = 2 of 18), and no identifiable mechanism (6%, *n* = 1 of 18) (Harada *et al.* 2022). Among patients with on-target resistance, the most common mutation involved the solvent front (87%, *n* = 13 of 15: *n* = 7 *NTRK3 G623R*, *n* = 4 *NTRK1 G595R*, *n* = 1 *NTRK2 G639L*, *n* = 1 *NTRK3 G623E*) followed by the gatekeeper region (13%, *n* = 2 of 15: *n* = 1 *NTRK1 F589L*, *n* = 1 *NTRK3 F617I*). Two patients developed off-target alterations including a *BRAF V600E* mutation and a *MET* amplification.

Second-generation NTRK inhibitors with clinical activity against resistance mutations, such as selitrectinib (NCT03215511) and repotrectinib (NCT03093116), are currently being explored in separate phase 1/2 trials.

## Clinical considerations for treatment with KIs in thyroid cancers

KIs have revolutionized the treatment of thyroid cancers. However, these treatments are not without significant issues including a challenging side-effect profile and the emergence of drug-resistant clones with treatment. In an open-label phase 2 trial of patients with *BRAF-*driven solid tumors that included 36 patients with ATC, the combination of BRAF and MEK inhibition with dabrafenib and trametinib was associated with treatment-related serious AEs in 19% (7 of 36). Grade 3/4 AEs were seen in 58% (21 of 36) with anemia (19%) and pneumonia (19%) being the two most common (Subbiah *et al.* 2022). Pyrexia occurred in 47% of patients, requiring the skillful use of steroids and treatment delays and/or dose reductions. Caution has to be used in patients with heart failure with reduced ejection fraction as there is a potential for cardiomyopathy with MEK inhibitors. A baseline echocardiogram and repeat echocardiograms every 3 months are warranted. Other rare but noteworthy adverse effects include uveitis and hemorrhage.

KIs that target VEGFR have unique adverse effects such as hypertension, hemorrhage, cardiovascular thrombosis, proteinuria, hand-foot syndrome, and fistulas. Patients with a predisposition to these conditions need to be screened out appropriately. A meta-analysis of trials of KIs in thyroid cancers revealed that the discontinuation rate was similar for sorafenib (16%), lenvatinib (16%), and vandetanib (19%). Sorafenib had increased rates of grade 3/4 hand–foot skin-related toxicities (21%) and rash (6%). Lenvatinib was associated with grade 3/4 hypertension in 28% of patients, and 3% had grade 3/4 proteinuria. The incidence of deaths caused by fatal AEs associated with each KI was 0.7, 1.9, and 1.7% for sorafenib, lenvatinib, and vandetanib, respectively (Oba *et al.* 2020). The newer-generation selective inhibitors of RET – selpercatinib and pralsetinib – have relatively lower rates of treatment-related grade 3 or higher AEs: 28 and 54%, respectively (Subbiah *et al.* 2021*a*). A baseline EKG and close monitoring of QTc is imperative for patients on vandetanib and selpercatinib due to the risk of QTc prolongation. Treatment with vandetanib also requires educating patients on strategies to limit UV exposure to limit photosensitivity reactions.

The risks and attendant toxicities associated with VEGFR KIs pose difficulties in deciding when to start KI therapy in patients with RAI-R DTCs. Some patients have relatively SD for prolonged periods and starting a KI in these patients may have an unacceptable risk/benefit profile. A post-hoc analysis was performed to assess if there was a differential benefit in OS based on baseline tumor burden in patients treated with lenvatinib from the SELECT trial (Kiyota *et al.* 2022). In an unadjusted analysis in the subgroup of patients with baseline sums > 4 cm (*n* = 182), there was seemingly no improvement in survival with lenvatinib when compared to placebo (29.1 vs 31.6 months, HR = 1.07; 95% CI, 0.78–1.48). However, to account for the high rate of early crossover from placebo to lenvatinib in the high tumor burden group, an adjusted rank-preserving structural failure time model analysis was performed; patients with high baseline tumor burden who received lenvatinib were also confirmed to have increased survival vs those who received placebo (29.1 vs 14.3 months, HR = 0.49; 95% CI, 0.30–0.74).

Patients with slow-growing diseases may end up needing to take these medications for prolonged periods of time, thereby exposing them to a higher risk of side effects and financial toxicity. Therefore, VEGFR KIs are currently recommended in cases with an increased rate of tumor burden growth or cases in which a high tumor burden portends a high risk of poor overall prognosis. The availability of multiple systemic options in patients with molecular drivers of disease such as *RET, BRAF,* and* NTRK* alterations poses a question of how best to sequence these modalities. This remains an open question of active investigation, which can be best answered with randomized clinical trials. A general approach for agent selection should aim to individualize decision-making after considering the risks and benefits in each situation.

## Conclusions and future perspectives

The advent of KIs targeting angiogenesis and MAPK signaling has changed the treatment paradigm for thyroid cancers. Sorafenib, a KI with activity against MAPK and VEGFR signaling, was the first KI approved for RAI-R DTC, which was followed by the validation and approval of lenvatinib and cabozantinib. Vandetanib and cabozantinib are multikinase inhibitors that are approved for advanced MTC. In PTCs and MTCs with driver *RET* alterations, the advent of potent and selective RET inhibitors, such as selpercatinib and pralsetinib, has led to substantial increases in efficacy and quality of life with limited toxicity. *NTRK*-driven thyroid cancers are highly sensitive to KIs such as larotrectinib and entrectinib. There is evidence that selective KIs that target BRAF, MEK, RET, NTRK, and ALK can re-sensitize refractory DTCs to take up RAI. The outcomes of ATC patients with *BRAF V600E/K* mutations have improved significantly with combination strategies utilizing BRAF and MEK inhibition. Emerging evidence also indicates that the combination of lenvatinib with the immune-checkpoint inhibitor pembrolizumab shows significant synergy in ATC.

Outstanding questions in the field include the optimal strategy of sequencing different therapies, especially in patients with driver *BRAF*-mutated tumors who have the added option of agents targeting the MAPK pathway in addition to the anti-angiogenic agents. New targeted agents that can inhibit bypass signaling through *RAS* mutations and RAC1 activation may help combat resistance to BRAF/MEK inhibition. Novel next-generation RET and NTRK targeted agents that have sensitivity to solvent-front resistance mutations and gatekeeper mutations are in early-phase trials and hold promise for patients with resistance that is driven by these alterations. Ongoing trials of note are summarized in Table 3.

## Declaration of interest

MS declares research funding from Merck and Eli-Lilly. BK declares research funding (to institution) from Eli Lilly & Co, Merck, Eisai, and Xencor. VS declares research funding from Eli Lilly & Co.

## Funding

This review was not funded by any entities.

## Author contribution statement

PJ and VS contributed to manuscript writing and editing. BK and MS contributed to manuscript content and editing. All authors contributed to the article and approved the submitted version.
